# Determining patient activity goals and their fulfillment following total knee arthroplasty: Findings from the prospective, observational *SuPeR Knee* study

**DOI:** 10.1371/journal.pone.0317205

**Published:** 2025-01-24

**Authors:** Karen Ribbons, Kristy Payne, Elizabeth Ditton, Sarah Johnson, Adrian Wills, Frederick Rohan Walker, Michael Pollack, Michael Nilsson

**Affiliations:** 1 Centre for Rehab Innovations, University of Newcastle, Callaghan, NSW, Australia; 2 Hunter Medical Research Institute, New Lambton Heights, NSW, Australia; 3 College of Health, Medicine and Wellbeing, University of Newcastle, Callaghan, NSW, Australia; 4 College of Science and Engineering, University of Newcastle, Callaghan, NSW, Australia; 5 Hunter New England Local Health District, John Hunter Hospital, New Lambton Heights, NSW, Australia; 6 Lee Kong Chian School of Medicine, Nanyang Technological University, Singapore, Singapore; Casa di Cura Villa Erbosa, ITALY

## Abstract

**Background:**

Dissatisfaction with Total Knee Arthroplasty (TKA) surgical outcomes remains between 10–20% and is associated with higher levels of societal costs. Expectations regarding post-surgical outcomes is considered as one of the major factors influencing satisfaction, however, there are no standardised methods for assessing patient’s expectations regarding activities to be achieved following surgery.

**Objectives:**

The aims of this study were to identify patient expectations relating to activities of importance following TKA and to describe goal fulfillment at 3 months post-TKA. We hypothesised that activity expectation fulfillment would be associated with overall satisfaction with TKA outcomes.

**Methods:**

This study comprised secondary data analysis of findings from the SuPeR Knee study. Using conventional content analysis, a classification system of activities specific to our TKA patient cohort was created. At 3 months following TKA, patients rated satisfaction with fulfilling activity goals and pain attenuation. The average level of satisfaction achieved was used as our measure of goal fulfillment. Overall satisfaction of the outcomes of surgery was rated using a 5-point Likert scale and the association between goal fulfillment and overall surgery satisfaction was compared by Spearman’s rank correlation.

**Results:**

Data were collected from 861 TKA patients. Recreation and sporting pursuits were found to be important activity types (43% of all activities). At 3 months after surgery, less impactful activities were more commonly satisfied (67%), including domestic and vocational activities, low impact hobbies and leisure activities. Goal fulfillment and improvement in knee pain were both significantly positively correlated to, and significant predictors of, overall patient satisfaction (p≤0.001).

**Conclusions:**

Our Australian cohort of TKA patients have a range of expectations for undertaking high-impact activities after surgery. However, at 3 months after surgery, higher rates of satisfaction were attained for lower-impact activities. Our findings support the importance of identifying activity expectations for each patient and that fulfillment of these goals contributes to overall satisfaction with the outcomes of TKA.

## Introduction

Total knee arthroplasty (TKA) is an effective treatment for end-stage osteoarthritis, resulting in improvement in joint function and attenuation in local pain, and with low rates of revision and mortality [1, 2]. These outcome measures are features considered important from a clinician’s perspective, but don’t address outcomes important to patients, which are more focused on achieving specific goals relating to knee function following TKA. Indeed, systematic reviews of satisfaction rates following TKA, consistently report that up to 20% of patients report dissatisfaction with surgical outcomes, often associated with a lack of improvement in knee function, range of motion and residual post-surgical pain [3–6].

As the number of TKA procedures is forecast to escalate globally over the next decade [7, 8], it is important to examine the causes of patient dissatisfaction, as this may impact on patient selection for surgery and improve clinical strategies to support or treat dissatisfied patients [9]. Moreover, it has been shown that dissatisfied patients have up to 57% greater societal costs, including patient-funded therapies, time away from paid employment and carer costs, 1 year following surgery compared to their satisfied counterparts [10].

Several pre-surgical patient features have been associated with dissatisfaction with surgical outcomes following TKA including pre-surgical knee symptomology [7, 11, 12] and psychological distress [6, 11, 13]

Expectation fulfillment has consistently been shown to be positively associated with satisfaction after TKA surgery [4, 6, 14, 15]. This is despite the fact that there is a large variety of ways to assess expectations and their fulfillment between studies, which can be a limitation in addressing expectation fulfillment in routine clinical practice. In some studies, expectations have been limited to achievement of outcomes, including attenuation of pain and functional items such as performance of usual daily activities and walking [14]. Standardized tools have also been developed which provide categorized expectations which are rated by importance prior to surgery and then the level of fulfillment post-TKA [16]. One concern regarding this approach is the categories generated may not have validity in different regional or cultural settings or reflect the type of expectations being set by younger patients undergoing TKA surgery. Given the projected increasing demand for TKA in younger patients of working age [17], the type and fulfillment of expectations may be evolving.

An alternative to providing patients with a standardized set of expectation categories is the adoption of an inductive content analysis approach, which facilitates the understanding of patient-specific expectation categories without the constraints imposed by pre-determined categories [18]. This methodology involves the use of open-ended questions to provide flexibility for patients to identify the expectations most meaningful to them thereby individualizing patient goals and goal fulfillment. With the increase in the number of TKA patients in paid employment, addressing expectations to achieve specific work-related activities [19, 20] may enhance interventions to improve outcomes and satisfaction.

While the level of overall satisfaction in TKA outcomes is comparable between patients in the USA, UK and Australia, Australian patients were more likely to expect better functioning at 12 months after surgery than their counterparts, and they also placed a greater importance on being able to undertake recreational activities and walk longer distances unaided [21]. This highlights the need to adopt methods which consider local cultural influences and individual variation regarding expectations of patient activities following TKA.

The purpose of this study was to describe and assess expectations relating to activities of most importance to a cohort of Australian TKA patients prior to surgery using a content analysis approach, to categorise these in a classification system, and to determine the level to which these expectations were fulfilled after surgery. We hypothesised that activity expectation fulfillment would be associated with overall satisfaction with TKA outcomes.

## Methods

The current study is a secondary analysis of prospectively collected data from the SuPeR Knee: Support, Predict, Recover study undertaken by the Centre for Rehab Innovations, NSW, Australia. The study was registered with Australian New Zealand Clinical Trials Registry (ANZCTR) 12619001000190; https://www.anzctr.org.au/ACTRN12619001000190.spx.

Universal Trials Number (UTN) U1111-1235-7747 and allocated the International Registered Report Identifier (IRRID): DERRI-10.2196/48801. The primary aim of the SuPeR Knee study was to investigate the predictive capacity of a comprehensive battery of biopsychosocial pre-surgical features on improvement in patient outcomes at 3 months after TKA [22]. Participant recruitment was commenced on 30^th^ October 2019 and completed on 30^th^ June 2022.

### Inclusion and exclusion criteria

Patients were included if they were over the age of 18, and identified as requiring unilateral or simultaneous bilateral total knee arthroplasty (TKA). Patients were excluded if knee arthroplasty was being undertaken due to trauma, they had undergone any knee surgery within the prior 6 months, or if they had knee surgery scheduled to take place within the following 12 months.

### Patient enrolment

Patients were referred for inclusion in the study by 11 orthopaedic specialists, with surgery being undertaken at one of four Ramsay Healthcare facilities in NSW, Australia including: Lake Macquarie Private Hospital, Gateshead; Baringa Hospital, Coffs Harbour; Kareena Hospital, Caringbah; and Wollongong Private Hospital, Wollongong. Study recruitment took place from November 2019 to June 2022. During this time, 1050 TKA patients were enrolled, with all patients providing written or electronic informed consent prior to study commencement.

### Ethical review

Research methodology was peer-reviewed and approved by the School of Medicine and Public Health at the University of Newcastle, NSW, Australia, in accordance with the Australian Code for the Responsible Conduct of Research. This study was conducted in accordance with the ethical standards in the 1964 Declaration of Helsinki and the National Statement on Ethical Conduct in Human Research (2007). Ethics approval was granted by the University of Newcastle Human Research Ethics Committee (approval H-2019-0109) on 21 June, 2019.

### Data collection and informed consent

Data collection occurred at two time points, during the month preceding scheduled TKA and at 3 months (12 weeks) following surgery. At each time point patients were asked to complete a series of study questionnaires. Participants could opt to access the questionnaires via a study-specific online portal using the web-based Visiontree Optimal Care platform (Visiontree San Diego, CA 92108, USA) accessed using a participant-specific login. Following review of the Patient Information Sheet, participants were asked to acknowledge their consent to participate in the study and provide an electronic consent on the Electronic Letter of Consent Form accessed after login. Questionnaire templates were not accessible by the participants until the consent field on this form had been completed. Alternatively, participants could request to have their study questionnaires posted to them, with a copy of the Patient Information Sheet and Letter of Consent included in the posted package. Participants were asked to provide written consent by signing the consent form prior to commencing completion of the questionnaires. The completed consent form and questionnaires were subsequently posted back to the study team.

Study participation was voluntary. Patients had the capacity to decline to participate at any time point during the study and were informed that this would not impact their ongoing medical care. If patients decided to withdraw from the study, they could specify if their data was to be removed from the study or if they were willing for it to be used as part of the analysis.

#### Expectation and satisfaction questions and responses

Data relating to patient expectations was collected during the month preceding TKA surgery with satisfaction of expectation fulfillment assessed at 3 months following TKA as described in Table 1. Prior to surgery, information was captured regarding patients’ expectations regarding activities they wanted to achieve post-TKA and the level of knee pain relief they envisaged following surgery. Patients were followed up at 3 months following surgery and were asked to indicate how satisfied they were that the activities they nominated prior to surgery could be undertaken, the level of knee pain attenuation experienced and their overall satisfaction with the outcomes of the surgery.

**Table 1:** Expectations and satisfaction questions.

Domain	Purpose	Question	Type	Response Options	Assessment timepoint
**Activity Expectations**	Assess the participant’s expectation of activity-related goals	“What activity/ies are you hoping to be able to do after your knee surgery that you cannot do now? (for example, this could be an every-day life activity, employment or work activity, social activity, specific movement / motion or exercise etc.).”	Open-ended	Participants were asked to list up to 3 items in 3 blank (open-ended) text boxes provided in the questionnaire.	Pre-TKA
**Pain Expectations**	Assess the level of expectation regarding alleviation of pain from the participant’s operated knee	“Thinking about three months after your surgery, do you expect the pain in your operated knee to be…”	Closed response	Participants provided a response on a 7-point Likert scale, ranging from 1 (very much worse) to 7(very much improved).	Pre-TKA
**Expectation Fulfilment**	**Activity Expectations:** Assess the participant’s level of satisfaction with their ability to undertake each of the activities identified as important to them prior to their TKA	Participants were presented with each of the expectations they identified pre-TKA assessment (Activity Expectations) and were asked to rate how satisfied they were that each activity could be undertaken	Closed response	Participants provided a response on a 7-point Likert scale ranging from completely dissatisfied (1) to completely satisfied (7)	3 months Post-TKA
	**Pain Expectations:** Assess the participant’s perception of the change in their knee pain following the TKA, relative to their pain pre-TKA	“The pain in my knee is now ….”	Closed response	Participants provided a response on a 7-point Likert scale, ranging from 1 (very much worse) to 7(very much improved).	3 months Post-TKA
**Overall Satisfaction**	Assess the level of overall satisfaction our study cohort had with their TKA	“Overall, how would you describe the results of your operation?”	Closed Response	Participants provided a response on a 5-point Likert scale, ranging from 1 (poor) to 5 (excellent).	3 months Post-TKA

### Data analysis

#### Classification of activities

The data used to classify activities were from participants who completed at least one text field in the pre-TKA questionnaire, describing an activity expectation, and this activity had a corresponding rating of expectation activity satisfaction at 3 months after TKA. Responses containing multiple activities in the same text field in the pre-TKA questionnaire were excluded from the analysis to avoid ambiguity with interpretation of the satisfaction of expectation fulfillment rating.

A conventional approach to content analysis was undertaken by KR and KP to analyse open-ended text responses and to develop an activity classification model. Content analysis is widely used in health studies for the analysis of text data [23]. Conventional content analysis allows large amounts of data to be intensely examined and classified, into a smaller number of categories that represent similar meanings [24], with the coding categories being derived directly from the text data. An inductive approach to category development was taken by the researchers, allowing the classes to flow from the data [24], thereby avoiding imposition of preconceived categories on to participants [25]. To ensure rigour of the analysis, the text data derived from 200 participants were read by both researchers independently. The researchers then highlighted text that described activities and derived initial code names to represent these. These activity descriptions and code names were subsequently discussed and compared by KR and KP and a consensus was reached regarding preliminary activity codes. The process of independent code generation followed by discussion, comparison, and challenging individual assumptions between the two researchers was utilised to decrease bias in the subjective interpretation of the data. These codes were sorted into higher level categories and sub-categories, with definitions for each specified, resulting in a preliminary classification system. Using an iterative process, the data from the original coded set of 200 and from the remaining participants were equally divided between KR and KP and coded (or re-coded in the case of the original data set) independently using the classification system. KR and KP discussed any data for which the preliminary codes were not appropriate and added new codes accordingly. A third researcher and subject matter expert (MP) reviewed and verified codes in the classification system in an iterative process. All coded data were then combined and compared by KR and KP and, using WHO ICF classifications [26] and terminologies as a reference, final activity classes with a numeric coding scheme were named, resulting in the classification system with major classes and subclasses as shown in Table 2. Frequency counts were generated for the number of “mentions” in the data for each major- and/or sub-class within the classification system.

**Table 2:** Distribution of activities identified.

Activity Class	Number	% of Total
**Total number of activities reported pre-surgery**	1886	
**0 -Pain—**Pain-free (not associated with a specific activity, “same activities without pain”).	10	0.5%
**1—Impairment**		
***1*.*1 Movement***—balance, bend, kneel, leg movement, stand, sitting, any knee movement, stairs, stability.	197	10%
***1*.*2 Sleep***–to encompass any mention of sleep	8	0.5%
**2—Activity Limitation**		
***2*.*1 Mobility***–walking (longer, further, faster, go for a walk, go walking, treadmill, up/down hill, uneven ground), driving a vehicle.	342	18%
***2*.*2 Personal ADLs / Transfers–***Arise from a chair, Get in and out of a bath, Get in and out of car, Get on and off a bus, Get on and off the toilet, Getting down and arise from the floor.	51	3%
***2*.*3 Normal life***—to encompass activity descriptions like "getting back to normal" or "get on with things".	32	2%
**3—Instrumental Activities of Daily Living**		
***3*.*1 Domestic / Vocational—***Domestic activity (housework, gardening, shopping). Work/occupational activity/volunteer work, lifting, climbing ladders.	301	16%
**4—Participation Restriction**		
***4*.*1 Social and Inter-personal—***socialising, helping others, family interaction, looking after/playing with grandchildren.	87	5%
***4*.*2 Leisure and Hobbies—***Camping / Caravaning, travel / holidays, beach/picnic/outdoor leisure, play cards/craft/photography, choir.	59	3%
**5—Recreation and Sport**		
***5*.*1 Active recreation—***bowls, golf (golf course walking), bike riding, acquarobics / hydrotherapy, pool/swimming, walking (for exercise, the dog, long distance, walking group, walk with friends), fishing, boating, canoeing/kayaking, yoga, exercise, gym.	563	30%
***5*.*2 Impact activities—***cricket, hockey, tennis, squash, running, karate, rock climbing, hiking / walking more than 5km / beach walking, dancing, surfing, snow skiing, horse riding.	236	13%

#### Association between expectation fulfillment and overall satisfaction

The relationship between satisfaction with expectation fulfillment and overall procedure satisfaction was explored using correlation analysis. Firstly, a new variable was created for the average expectation fulfillment satisfaction rating for each participant. This was derived by viewing the satisfaction ratings provided by each participant for all activities listed prior to surgery and calculating the average level of satisfaction achieved across all activities, which was defined as their level of activity expectation fulfillment satisfaction. Patients with missing data were removed from the analysis. Non-parametric methods were used due to the ordinal nature of the data. Therefore, the relationship between activity expectation fulfillment satisfaction and overall procedure satisfaction was examined using Spearman’s rank correlation coefficient. Similarly, the association between the level of improvement in knee pain and overall satisfaction was compared using non-parametric analysis (Spearman’s rank correlation).

## Results

### Responses received and evaluable data

Of the 1050 TKA patients who consented to participate in the SuPeR Knee study, 974 participants completed at least one of three text boxes for activities to be improved by surgery. Of these, 861 participants completed satisfaction rating for activities being fulfilled at 3 months post-surgery. The number of participants who completed expectation and satisfaction ratings specifically relating to knee pain (in the closed-response questions) was 854.

The number of activities specified by patients was varied within the study cohort. Some participants provided one activity, while others provided 3 activities. Rather than selecting specific activities from each patient in our analysis, we combined the activities across all participants, such that our analysis examined expectation and satisfaction across the whole study cohort rather than on an individual patient basis. There were 124 text boxes that were excluded from the analyses, as they contained multiple activities and there was no way of determining which of these the participant was rating for satisfaction.

In total, 1886 evaluable activities and corresponding satisfaction ratings were derived from our study cohort and used in the current analysis.

### Cohort characteristics

The average age of study participants was 68 +/- 8 years and was comprised of 49.8% female patients. Patients from each of the 4 participating hospitals were included in the evaluable dataset. The majority (87%) underwent a unilateral TKA, with a primary diagnosis of osteoarthritis (99%).

### Activity expectations

SuPeR Knee study participants (n = 861) provided a total of 1886 activities they were hoping to be able to achieve following their TKA. The frequency of each activity class is shown in Table 2 along with items that were included in each class. The most frequently mentioned activities were those in Category 5 (Recreation and Sport), accounting for 43% of all activities listed prior to surgery.

Active recreation activities (5.1), including walking for exercise, bike riding and swimming, represented 30% of all tasks indicated. The least mentioned activities included Category 0 (Pain) and Category 1.2 (Sleep).

### Satisfaction with expectation fulfillment

**Fig 1:**
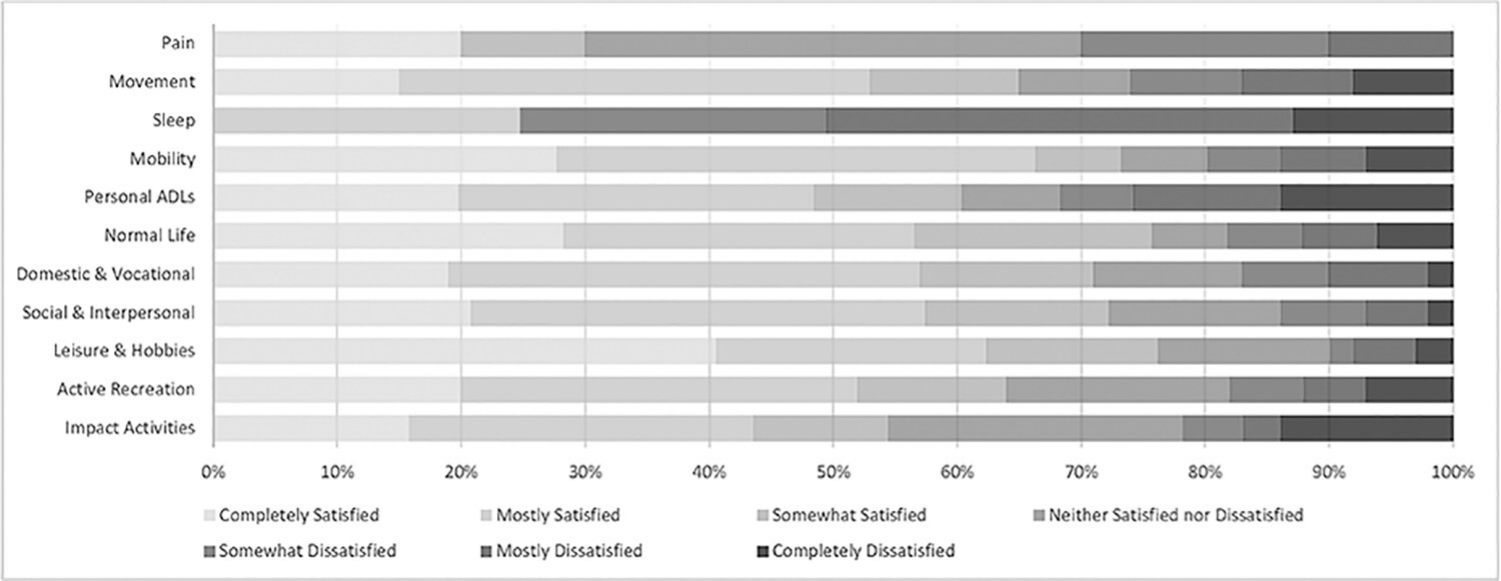
Frequency of satisfaction ratings for each activity class.

For each activity class, the frequency of each satisfaction rating for the activity expectations to be fulfilled at 3 months after TKA is shown in Fig 1. Higher levels of dissatisfaction were found for achieving recreation and sport impact activities and personal daily living activities (ADLs). In contrast, higher levels of satisfaction were reported for lower impact activities including domestical and vocational pursuits as well as social and leisure activities.

#### Satisfaction summary

We combined the frequency of the above ‘Completely Satisfied’ and ‘Mostly Satisfied’ ratings to provide a summary metric of satisfaction (labelled ‘satisfied’) with activity fulfillment post-TKA. This provides a summary frequency measure for the outcomes of the TKA improving participants’ ability to undertake each activity class as shown in Table 3.

**Table 3:** Expectation fulfillment satisfaction rates per activity class.

ACTIVITY CLASS	Satisfied
**0—Pain**	20%
**1—Impairment**	
***1*.*1 Movement***	53%
***1*.*2 Sleep***	25%
**2—Activity Limitation**	
***2*.*1 Mobility***	67%
***2*.*2 Personal ADLs / Transfers***	49%
***2*.*3 Normal life***	56%
**3—Instrumental Activities of Daily Living**	
***3*.*1 Domestic / Vocational***	57%
**4—Participation Restriction**	
***4*.*1 Social and Inter-personal***	58%
***4*.*2 Leisure and Hobbies***	63%
**5—Recreation and Sport**	
***5*.*1 Active recreation***	52%
***5*.*2 Impact activities***	44%

The highest rate of satisfaction for expectation fulfillment was achieved for improvement in Category 2.1 Activity Limitation activities (including walking and driving a vehicle), with over 2/3 of responses being associated with a ‘satisfied’ rating at 3 months following their TKA. Participation Restrictions (Category 4) had the second highest level of satisfaction, with 63% of ‘leisure and hobbies’ activities having a ‘satisfied’ rating allocated to them by participants (Category 4.2).

Although Recreation and Sport (Category 5 activities) were the most frequently mentioned activities participants wanted to be improved by their TKA, at 3 months post-surgery only 48% of such activities were associated with a satisfaction rating post-surgery (including playing bowls, golf and walking for exercise).

### Overall TKA satisfaction

**Fig 2:**
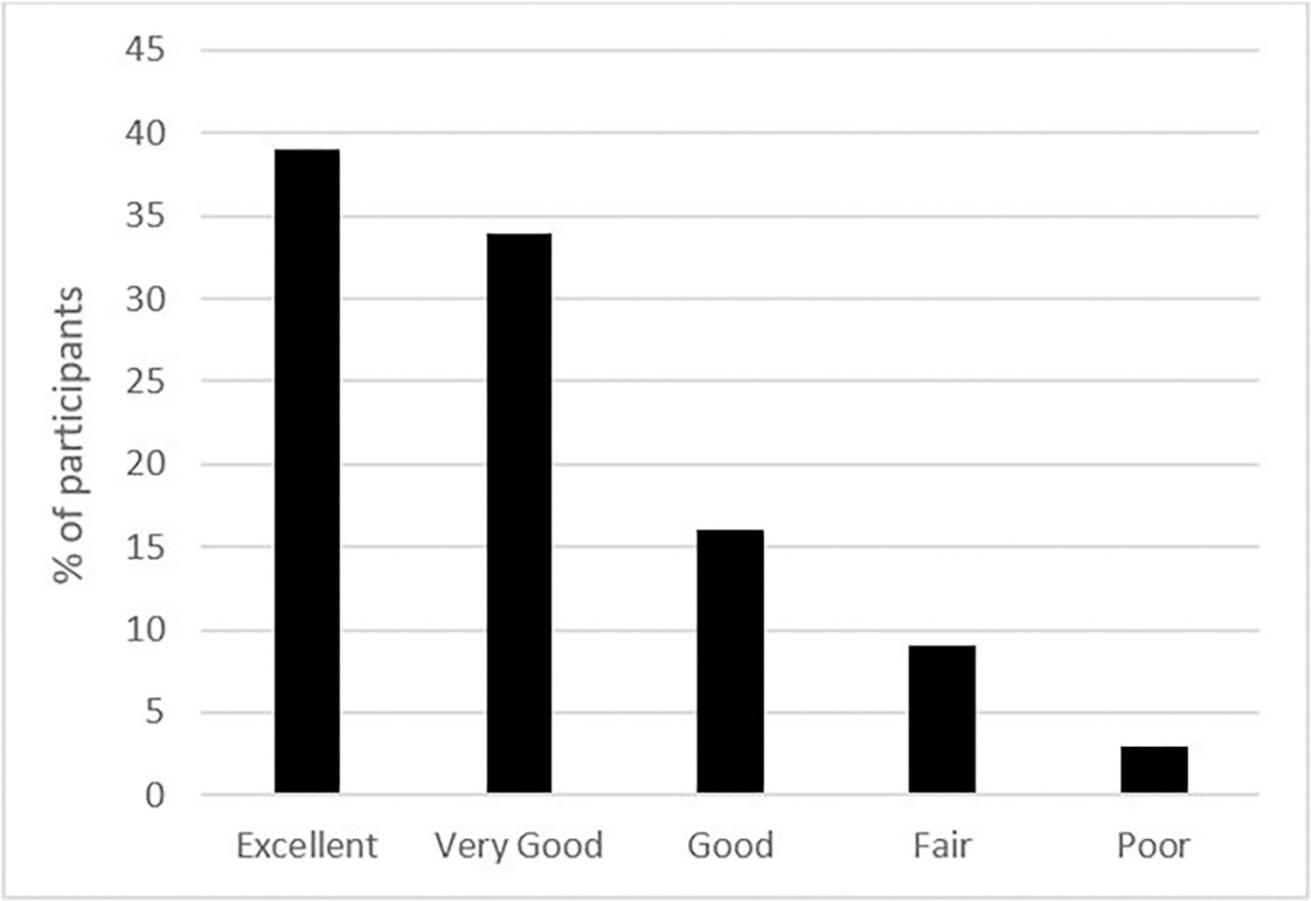
Overall satisfaction with outcomes of the surgery 3 months post-TKA.

The level of overall satisfaction with the TKA at 3 months after surgery was high, with 89% of respondents providing good to excellent ratings for the TKA (Fig 2).

### Pain-specific expectation and satisfaction

**Fig 3:**
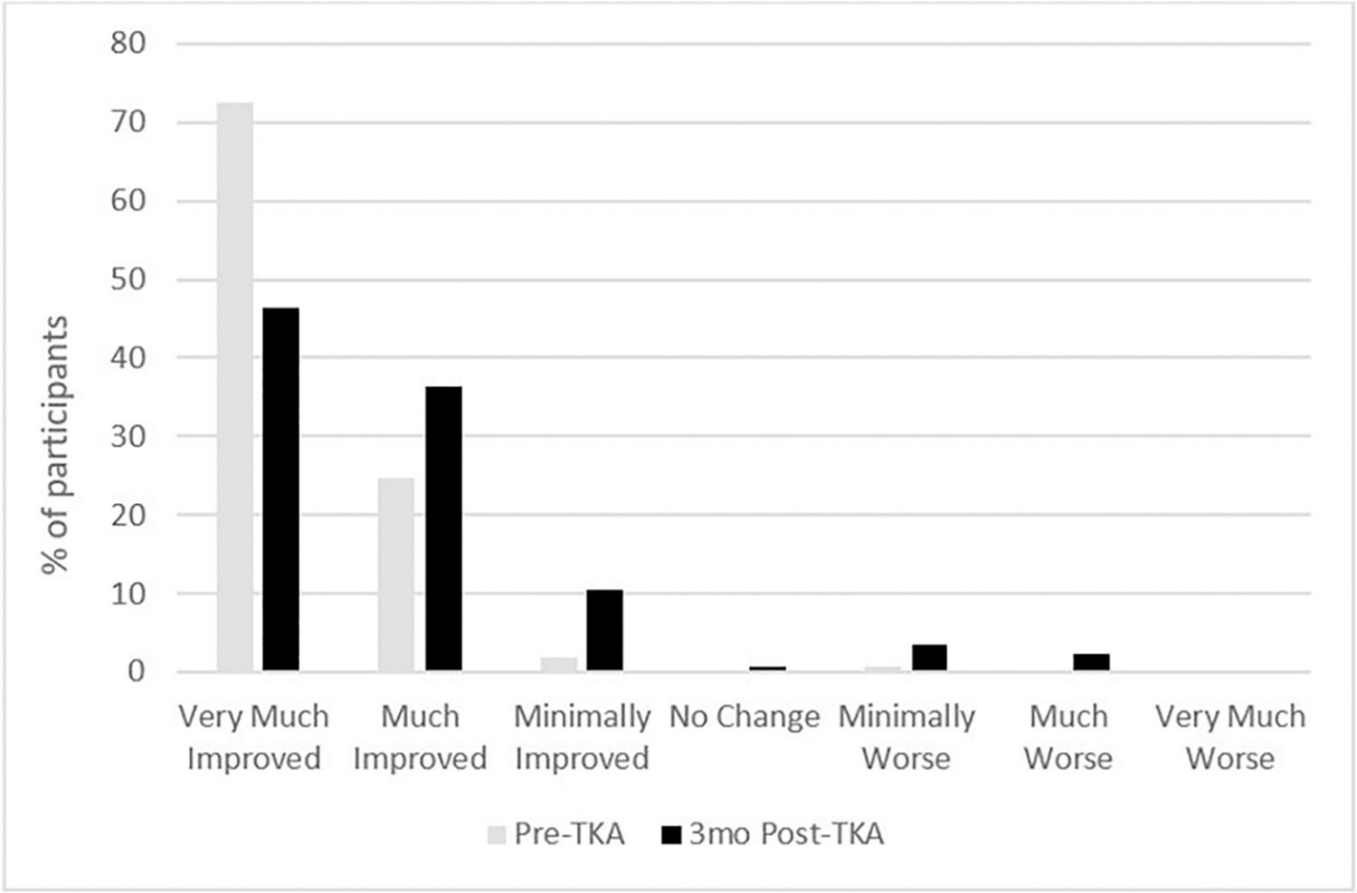
Expected reduction in knee pain pre-surgery and reported knee pain reduction 3 months post TKA.

The distribution and frequency of expectation and attenuation ratings relating to knee pain is summarised in Fig 3 (n = 854). Prior to the TKA, 97% of our study cohort expected knee pain to be very much or much improved by the surgery. However, at 3 months after TKA, knee pain attenuation levels were lower than the expectation levels expressed. Pre-TKA, 72% expected knee pain to be very much improved, while at 3 months post-TKA 46% indicated knee pain being very much improved. Furthermore, 6% of respondents indicated worsening knee pain at 3 months post-surgery.

### Association between expectation fulfillment and overall satisfaction

There were 845 patients who had completed a rating for overall satisfaction, activity expectation fulfillment satisfaction, and level of pain improvement and were included in the analysis. For satisfaction with activity expectation fulfillment the single summary ‘satisfaction’ metric was used. A significant moderate positive correlation between overall satisfaction with surgery outcomes and satisfaction with activity expectation fulfillment was found, *r*(845) = 0.57 p≤0.0001. We also compared the level of improvement with knee pain at 3 months post-surgery with the overall satisfaction with surgery outcomes and found a significant positive correlation between the two parameters, *r*(845) = 0.67 p≤0.0001.

## Discussion

Our study is unique in that we evaluated post-TKA satisfaction in relation to responses to an open-ended question about expectations, with participants able to specify these using their own words. Few prior studies have used open-ended questions to assess patient expectations as a routine clinical approach. However, this approach enables responses to be individualized for each patient and enables lifestyle and social factors to be considered when evaluating patients’ expectations regarding TKA outcomes. Furthermore, our study focussed on participants’ expectations regarding their ability to perform specific activities rather than the more general expectations that have been evaluated previously. In our patient cohort we identified active recreational pursuits as the most common activities wanting to be achieved following their knee arthroplasty. However, at 3 months following surgery less than half of the participants were satisfied with their ability to achieve these goals. Both satisfaction with achieving activity goals and the level of pain attenuation achieved, were associated with overall satisfaction with surgical outcomes. This supports our hypothesis that activity expectation fulfillment was associated with overall satisfaction.

Using an inductive content analysis approach we identified a total of 6 major classes of activities TKA patients would like to achieve after surgery. The activities ranged from knee movement impairment-specific tasks, such as sitting or standing, to high-impact sporting activities, including running and surfing. Our expectation classification contained fewer overall categories in comparison with other TKA studies. For example, Mancuso et al. 2001 [16] found 20 expectation classes and Conner-Spady et al. 2020 [18] found 24. Although our study identified 13 activity types common to each of these studies, the categories found in these other studies extend beyond “activities”. Such studies have elicited expectations related to improvements in general health, avoidance of future knee degeneration [16]; quality of life, use of medications, sexual activity, use of walking aids, weight loss, impact on other joints [18]; and psychological well-being [16, 18]. These differences likely represent the more general nature of the question posed to participants in previous studies. Our study also classified activities in a slightly different way. For example, in the Hospital for Special Surgery Knee Replacement Expectations Survey (HSS) developed by Mancuso et al., 2001 [16] from a cohort of North American patients, items relating to knee impairment were each given a distinct class, whereas our study pooled these items into the same expectation class of ‘Impairment—Movement’. Our study, however, makes other important distinctions, such as between active recreation and impact activities, with the former appearing more frequently as an expectation in our study than the latter, whereas Mancuso et al., 2001 [16] grouped exercise and sports together in one category. Such groupings likely have a direct impact on the range of responses derived and implications for the way in which patients are asked about their expectations in the clinical setting. Indeed, when patients are provided with a list of impairments and asked to rate these in terms of importance and significance as a goal to be achieved [27], recreation and sports are not identified as main goals, but expectations related to pain, range of motion, walking distance, overall physical function, climbing stairs, quality of life, and implant survival are endorsed. This suggests that a pre-specified list of expectations may not elicit the range of patient-specific priorities that would otherwise be obtained when prompts are not used. On the other hand, when only asking about “activities”, other priorities may be missed. For example, pain-relief was mentioned infrequently (less than 1%) in response to the open-ended question about activities in our study, but we acknowledge that pain is important. Indeed, when we asked specifically about pain, 97% of respondents expected pain to be very much- or much-improved by surgery. Similarly, it has been found to be very important in other studies, when asking a general question about outcomes (rather than asking about activities) or when asking specifically about pain [18, 27–30]. When patients are asked a more general question about expectations, responses will likely include a greater range of categories, whereas asking about activities may better distinguish between the types of activities expected to be improved, particularly in relation to sport and recreation, but may miss other categories of importance. The advantage of the current approach, however, has the potential to allow patients to move beyond a pain focus, which can be limiting, and allow clinicians to support them in the implementation of practical, actionable, and achievable tasks that support recovery more broadly. In a clinical setting, it is likely that pain will be enquired about more routinely in a formal way post-surgically, while providing a framework for identifying and connecting patients with other personally meaningful activities might not be. While standardizing collection of expectations using validated questionnaires may be advantageous in the research setting the approaches taken in the current study explore the way to collect expectation information that has the greatest clinical utility to enable personalized, targeted discussions regarding patient expectations to be routinely performed.

The most common activities our Australian cohort hoped to be improved by TKA were active recreational and high-impact sporting activities (43%). This is consistent with expectations found in other studies using open-ended questioning. For example, among a North American orthopaedic patient cohort, return to sports was commonly expected, with a large range of sports anticipated, including those of high impact [31]. In our study the next most frequent category of activity was improvement in daily limitations of mobility, including walking and driving, with around one fifth of expected activity improvements falling into this category. Improved walking ability is consistently found to be important for TKA patients, including in North American, Canadian, Scottish, German, and Korean cohorts [16, 18, 27, 29, 30, 32], with up to 97% of those surveyed saying this is very important [32]. Our Australian cohort also hoped to see improvements in instrumental activities of daily living, including undertaking domestic and vocational work, which is similarly important to Canadian TKA patients [18, 32]. While elements of movement, such as ascending stairs and “range of motion” have been found to be included in the top priorities of cohorts in other studies [18, 27, 32], these were found at a somewhat low frequency (10%) in our study. So too, quality of life (encompassing elements such as physical, psychological, and social wellbeing; social participation; and independence) has been found to be important among other cohorts of TKA patients [27]. While we found that social and interpersonal participation and what may be considered elements of physical wellbeing (across activity types) are important, we did not elicit responses from participants on psychological wellbeing. It may be the case that differences in the top expectations identified between studies are reflective of regional and cultural variations in activity expectations. This supports the findings by Lingard et al., 2006 [21], which indicated a higher level of knee function and recreational activities is expected by Australian TKA patients compared with their USA and UK counterparts. Our findings reinforce the concept of addressing activity expectations important to patients prior to surgery at the local level. Our approach allows finer-grained detection of what is important for a specific cohort, whereas a categorical approach risks making assumptions about what will be important and may miss culturally-specific activities.

Expectations relating to mobility (e.g., walking and driving) were the most frequently met (67%) in our study, followed by leisure and hobbies (63%). The most common pre-surgical expectation (to undertake active recreational and sporting activities) was satisfied for just over half of the cohort, suggesting that for many TKA patients these more active pursuits remain unmet within this timeframe. This is consistent with previous research demonstrating that, between 6- and 12 months after TKA, fulfillment of expectations relating to physical activity and physical function is conservative at between 42 [33] and 68% [18]. Despite the nature of physical activities not being stated in these studies, taken together, these findings suggest that expectations relating to physical function may be unrealistic for some patients, and that returning to low-impact activities may be more likely [34]. In our study we were able to provide a more granular view of specific activities important to patients, and expectations about achieving these. Applying this approach to the clinical setting has the potential to enhance discussions regarding realistic goal setting with patients prior to their TKA with respect to achieving activity-related goals. Indeed, providing greater insight regarding achievable activity goals following TKA will potentially enable patients to make a better-informed decision regarding undertaking knee surgery. Such discussions are an avenue for clinicians to support patients to modify unrealistic expectations, with education programs directed at providing information relating to realistic goal-setting prior to TKA successfully altering patients perceptions regarding what can be achieved following surgery, which can in turn lead to increased satisfaction with surgical outcomes [35, 36].

Our closed-response pre-surgery question asking specifically about pain, indicated that 97% of the cohort expected pain to be very much or much improved after surgery and 82% found that it was indeed very much or much improved post-surgery. These findings are suggestive of higher levels of pain expectation satisfaction in our Australian cohort than in previous studies undertaken in other geographical locations. In a study using the HSS questionnaire, pain relief was considered the most important expectation for Scottish TKA patients [36], although at 6 weeks after TKA only 32% reported fulfillment of this expectation, which increased to 50% at 12 months [37]. The expectation with the highest level of complete fulfillment was knee and leg straightening seen in 58% of patients and this increased to 78% at 12 months after TKA [37]. Pain relief was also considered one of the most important expectations in a cohort of German patients, with 79% of patients indicating that this expectation was fulfilled or exceeded at 12 months after TKA [33].

In our study cohort, 11% of patients reported overall dissatisfaction with TKA outcomes, which is at the lower end of the range commonly reported (10–20%) [4, 6, 38]. Our study is important in that we found that satisfaction with activity expectation fulfillment and improvement in knee pain attenuation were associated with patient’s overall satisfaction rating of surgical outcomes. These findings are supported by several systematic reviews [4, 14] and in the guest editorial by Verhaar, 2020 [9], who concluded that expectation fulfillment is positively associated with overall satisfaction after surgery, irrespective of study design or the patient group investigated. Knee pain improvement has also been associated with higher overall satisfaction post-TKA. Univariate analysis of pre- and post-surgical variables between satisfied and dissatisfied patients revealed that the magnitude of change in self-reported pain levels was greater in satisfied compared to dissatisfied patients [39]. Indeed, residual knee pain is a common feature in dissatisfied patients [12]. These findings provide clinicians with a greater understanding of factors related to overall satisfaction with TKA and are suggestive of the importance of supporting patients to achieve fulfillment of patient-identified activities for improving rates of overall satisfaction with surgery. This requires discussions with patients, prior to surgery, to identify expectations in line with each patient’s individual priorities. Such discussions also present an opportunity to tailor rehabilitation and recovery efforts in ways that are more patient-centric and meaningful. Some of the variation in overall satisfaction ratings among TKA patients was not explained by our study. Even though expectation fulfillment may be linked to overall TKA satisfaction, the maximum level of satisfaction with activity expectations we achieved was 67%, while the overall dissatisfaction with TKA outcomes was low, even at 3 months after surgery. This suggests that incomplete expectation fulfillment can still be associated with high levels of overall patient satisfaction. This aligns with a recent study conducted with a cohort of UK patients [37], where the rate of overall expectation fulfillment was 48% at 12 months following TKA, while overall satisfaction with surgery outcomes was 89%. This leads us to question the clinical validity of asking patients if they are satisfied with their overall surgical outcomes. Ring and Leopold, 2015 [40] questioned the usefulness of addressing satisfaction following surgery as a single metric, due the complexities of outcomes and influences a patient must weigh-up in considering their response. It is also highly likely that other factors are contributing to patients’ views on overall TKA satisfaction and requires consideration when understanding patient satisfaction. Indeed, residual pain and less improvement in knee symptomology and issues with wound healing, have all been linked to dissatisfaction with surgical outcomes [5, 12]. In addition, factors prior to surgery including low quality of life and psychological distress have been identified as predictive factors for dissatisfaction with TKA outcomes [4, 5, 12, 41]. One possible approach to address patient satisfaction may be to question satisfaction in attaining both specific clinical outcomes and patient-specific goals. Insights gained from such questioning will inform ways to address patient-specific issues and facilitate education and treatment strategies better tailored to meaningful recovery outcomes. This approach could also facilitate a more individualized approach to rehabilitation which in turn could improve outcomes and overall satisfaction.

### Study strengths and limitations

The major strength of our study was using a content analysis approach to capture information regarding expectations relating to activities that patients wanted to achieve following their TKA. The classification of activities identified is far more granular regarding physical activities than those appearing in standardized tools for deriving information regarding patient expectations. We acknowledge that the content analysis approach is more time consuming than using standardized questionnaires to capture information regarding patient expectations and that the collection of free text does not allow for automated scoring systems to be applied. However, in the current study this procedure enabled the scope of activities that were important to our study cohort to be identified. In the clinical setting we would not intend for a full qualitative classification process to be undertaken as performed in the current study rather, we would envisage that identifying expectations important to individual patients is a simple technique which offers the potential to provide clinically meaningful, useful, and actionable information.

The need to pool activity data across our study cohort limited our capacity to make associations of activity types and expectation fulfillment with other individual patient biopsychosocial factors collected in the main study. In addition, we were not able to include the impact of surgical factors on activity expectation fulfillment as this information was not accessible for collection by the research team. Indeed, others have explored the potential impact of different implants on activity resumption and return to sports post-surgery [42], however found no difference in the impact of implant type on knee function and return to activities at 5 years post-TKA. In our study we collected information regarding satisfaction with fulfillment of pre-TKA expectations and overall surgical outcomes at 3 months after the TKA which may be considered early in a patient’s post-surgery recovery journey. Most other reported studies examining expectation fulfillment and satisfaction have measured these parameters at 6 or 12 months or for longer follow up times [18, 33, 39, 43]. However, expectation fulfillment and satisfaction has been reported by others within the 6-month interval following TKA. It has been shown that expectation fulfilment can been achieved in nearly a third of participants at as early as 6 weeks post-TKA, with this level increasing further at 12 months following TKA [37]. Interestingly, it has also been reported that the level of overall satisfaction achieved at 3 months following TKA was comparable to that reported at 6 months (80%), with the level of satisfaction increasing further at 12 months post-surgery (90%) [44]. Indeed, even though it was beyond the scope of the current study, it would be of interest to explore satisfaction levels for activity expectation fulfillment at later times following TKA, to determine if these parameters change as physical recovery continues in future studies.

We acknowledge that patient factors other than expectation fulfillment may be contributing to patient satisfaction. The current study focussed on the influence of activity expectation fulfillment and pain attention on satisfaction. We have collected other patient data relating to a range of biopsychosocial patient features which may be contributing to overall satisfaction, however, we feel that that these associations are beyond the scope of the current study but are worthy of consideration in future investigations.

In considering the validity of our findings in the global context, the high proportion of patients we see in our Australian cohort who identified high impact recreational and sporting pursuits as important expectations may differ in other countries due to cultural and regional factors and indeed has been shown by others [21]. As such, these factors need to be considered when generalizing our findings in the broader context, but also supports the notion that providing open ended questions to accurately identify individuals’ expectations may be the preferred method to allow for local cultural and social factors.

## Conclusions

Discussions about expectations of what TKA surgery may achieve in relation to participation in various activities are important for managing the potential mismatch with satisfaction levels for such expectations being met post-surgery. Consideration of the approach to asking patients about their expectations is important for not limiting the range of expectations patients specify.

Eliciting patient-specific expectations may require changes to the way that anticipated TKA patients are interviewed, with pre-specified lists of expectations supplemented by open-ended questions that allow patients to specify their priorities in their own words being important. The way in which questions about expectations of surgery are asked has an impact on the responses of patients. Our recommendation would be for specific questions relating to activity goals be asked to each patient prior to surgery, which may assist in setting realistic expectations and enable a more individualized approach to capturing information regarding meaningful expectations to be adopted which can potentially impact on patient satisfaction.

Expectations prior to surgery can provide a reference point for rehabilitation goals post-surgery. For example, if a patient wants to get back to a certain activity but doesn’t engage in a targeted rehab that facilitates this, the failure may not be the surgery but the lack of appropriate bridging support to restore function. It could provide surgeons with opportunities to pre-emptively connect patients with tailored rehabilitation programs that prioritise specific patient rehabilitation targets, or referrals to specific allied healthcare professionals to optimise the chances of satisfaction and recovery of function in meaningful areas.

## Supporting information

S1 Checklist Completed STROBE checklist. (DOCX)
