# Supplementary material for: Determining patient activity goals and their fulfillment following total knee arthroplasty: Findings from the prospective, observational SuPeR Knee study
Source: PLoS One. 2025 Jan 24;20(1):e0317205. doi: 10.1371/journal.pone.0317205 (PMC11759989; doi:10.1371/journal.pone.0317205)
Supplement: S1 Checklist — (DOCX) [file pone.0317205.s001.docx]

STROBE Statement—checklist of items that should be included in reports of observational studies

|  | Item No. | Recommendation | *Page  No. | Relevant text from manuscript |
| --- | --- | --- | --- | --- |
| **Title and abstract** | 1 | (*a*) Indicate the study’s design with a commonly used term in the title or the abstract | 1 (Title) | We have indicated that this is an observational, prospective study in the Title. |
|  |  | (*b*) Provide in the abstract an informative and balanced summary of what was done and what was found | 2, 3 (Abstract) | See methods and results sections of the Abstract. |
| Introduction | | | |  |
| Background/rationale | 2 | Explain the scientific background and rationale for the investigation being reported | 4,5 (Introduction) | Relevant background relating to the study is provided which supports the rationale for the study. |
| Objectives | 3 | State specific objectives, including any prespecified hypotheses | 5,6 (study aims) | Aims and hypothesis to be tested are stated. |
| Methods | | | |  |
| Study design | 4 | Present key elements of study design early in the paper | 6 | Study design is indicated. |
| Setting | 5 | Describe the setting, locations, and relevant dates, including periods of recruitment, exposure, follow-up, and data collection | 6 | Location of enrolled participants is indicated. |
| Participants | 6 | (*a*) *Cohort study*—Give the eligibility criteria, and the sources and methods of selection of participants. Describe methods of follow-up  *Case-control study*—Give the eligibility criteria, and the sources and methods of case ascertainment and control selection. Give the rationale for the choice of cases and controls  *Cross-sectional study*—Give the eligibility criteria, and the sources and methods of selection of participants | 6 | Inclusion and exclusion criteria and patient enrolment shown. |
|  |  | (*b*) *Cohort study*—For matched studies, give matching criteria and number of exposed and unexposed  *Case-control study*—For matched studies, give matching criteria and the number of controls per case |  | N/A |
| Variables | 7 | Clearly define all outcomes, exposures, predictors, potential confounders, and effect modifiers. Give diagnostic criteria, if applicable | 8-12 | Data collected and data processing procedures are outlined. |
| Data sources/ measurement | 8* | For each variable of interest, give sources of data and details of methods of assessment (measurement). Describe comparability of assessment methods if there is more than one group | 8-12 | Methods used to collect data are indicated for each variable. |
| Bias | 9 | Describe any efforts to address potential sources of bias | 8-9 | The provision of open-ended and closed questions provided in the study supported limiting bias of the responses obtained and data collected in this study. |
| Study size | 10 | Explain how the study size was arrived at |  | As this was secondary analysis to the main study no power calculation was undertaken to determine study size in the sub-study described in this paper. |

*page number shown refers to final clean version of the manuscript Continued on next page

| Quantitative variables | 11 | Explain how quantitative variables were handled in the analyses. If applicable, describe which groupings were chosen and why | 12 | Quantification of expectation fulfilment and satisfaction ratings is provided. |
| --- | --- | --- | --- | --- |
| Statistical methods | 12 | (*a*) Describe all statistical methods, including those used to control for confounding | 12 | Statistical methodology is described. |
|  |  | (*b*) Describe any methods used to examine subgroups and interactions |  | N/A |
|  |  | (*c*) Explain how missing data were addressed | 13 | The evaluable data used in the current analyses is described. |
|  |  | (*d*) *Cohort study*—If applicable, explain how loss to follow-up was addressed  *Case-control study*—If applicable, explain how matching of cases and controls was addressed  *Cross-sectional study*—If applicable, describe analytical methods taking account of sampling strategy | 12 | Only participants with recorded expectation fulfilment information was included in the analysis. |
|  |  | (*e*) Describe any sensitivity analyses |  | N/A: No sensitivity analyses was undertaken. |
| Results | | | | |
| Participants | 13* | (a) Report numbers of individuals at each stage of study—eg numbers potentially eligible, examined for eligibility, confirmed eligible, included in the study, completing follow-up, and analysed | 13 | For this sub study to the main study the number of evaluable patient data available for each analyses undertaken is described. |
|  |  | (b) Give reasons for non-participation at each stage |  | Refer to the protocol paper for the main study for details of non-participation. |
|  |  | (c) Consider use of a flow diagram |  | Flow chart of full study participation are shown in the protocol paper cited in the current paper. |
| Descriptive data | 14* | (a) Give characteristics of study participants (eg demographic, clinical, social) and information on exposures and potential confounders | 13 | Cohort features are provided. |
|  |  | (b) Indicate number of participants with missing data for each variable of interest | 13 | The number of participants providing evaluable data used in the analyses is indicated. |
|  |  | (c) *Cohort study*—Summarise follow-up time (eg, average and total amount) | 6 | The follow up time is described. |
| Outcome data | 15* | *Cohort study*—Report numbers of outcome events or summary measures over time | 13 | Numbers of participants contributing to the data used in the current study are provided. |
|  |  | *Case-control study—*Report numbers in each exposure category, or summary measures of exposure |  |  |
|  |  | *Cross-sectional study—*Report numbers of outcome events or summary measures |  |  |
| Main results | 16 | (*a*) Give unadjusted estimates and, if applicable, confounder-adjusted estimates and their precision (eg, 95% confidence interval). Make clear which confounders were adjusted for and why they were included | 13-18 | Proportions of each activity category, pain attenuation and satisfaction ratings are provided. |
|  |  | (*b*) Report category boundaries when continuous variables were categorized |  | N/A |
|  |  | (*c*) If relevant, consider translating estimates of relative risk into absolute risk for a meaningful time period |  | N/A |

Continued on next page

| Other analyses | 17 | Report other analyses done—eg analyses of subgroups and interactions, and sensitivity analyses | 13-18 | All analyses undertaken are described in the Results section. |
| --- | --- | --- | --- | --- |
| Discussion | | | | |
| Key results | 18 | Summarise key results with reference to study objectives | 21-22 | The major study findings are described. |
| Limitations | 19 | Discuss limitations of the study, taking into account sources of potential bias or imprecision. Discuss both direction and magnitude of any potential bias | 28 | We have discussed the main study limitations and strengths. |
| Interpretation | 20 | Give a cautious overall interpretation of results considering objectives, limitations, multiplicity of analyses, results from similar studies, and other relevant evidence | 28-30 | Findings from the current study have been compared to findings from others cited in the literature. |
| Generalisability | 21 | Discuss the generalisability (external validity) of the study results | 28 | The generalisability of our findings is discussed in the Strengths and Limitations section of the Discussion. |
| Other information | |  | | |
| Funding | 22 | Give the source of funding and the role of the funders for the present study and, if applicable, for the original study on which the present article is based |  | The funding source and role of funders for the main and current study has been indicated to the journal publishers on the submission platform. |

*Give information separately for cases and controls in case-control studies and, if applicable, for exposed and unexposed groups in cohort and cross-sectional studies.

**Note:** An Explanation and Elaboration article discusses each checklist item and gives methodological background and published examples of transparent reporting. The STROBE checklist is best used in conjunction with this article (freely available on the Web sites of PLoS Medicine at http://www.plosmedicine.org/, Annals of Internal Medicine at http://www.annals.org/, and Epidemiology at http://www.epidem.com/). Information on the STROBE Initiative is available at www.strobe-statement.org.
